# Iron Status and *Helicobacter pylori* Infection in Symptomatic Children: An International Multi-Centered Study

**DOI:** 10.1371/journal.pone.0068833

**Published:** 2013-07-04

**Authors:** Dulciene Maria Magalhaes Queiroz, Paul R. Harris, Ian R. Sanderson, Henry J. Windle, Marjorie M. Walker, Andreia Maria Camargos Rocha, Gifone Aguiar Rocha, Simone Diniz Carvalho, Paulo Fernando Souto Bittencourt, Lucia Porto Fonseca de Castro, Andrea Villagrán, Carolina Serrano, Dermot Kelleher, Jean E. Crabtree

**Affiliations:** 1 Laboratory of Research in Bacteriology, Faculdade de Medicina, Universidade Federal de Minas Gerais, Belo Horizonte, Brazil; 2 Division of Pediatrics, Unit of Gastroenterology and Nutrition, School of Medicine, Pontificia Universidad Catolica de Chile, Santiago, Chile; 3 Blizard Institute, Barts and the London School of Medicine and Dentistry, Queen Mary, United Kingdom; 4 Institute of Molecular Medicine, Trinity Centre for Health Sciences, St Jameśs Hospital, Dublin, Ireland; 5 Centre for Pathology, Division of Medicine, Imperial College, London, United Kingdom; 6 Endoscopy Service, University Hospital, Universidade Federal de Minas Gerais, Belo Horizonte, Brazil; 7 Department of Pathology, Faculdade de Medicina, Universidade Federal de Minas Gerais, Belo Horizonte, Brazil; 8 Department of Clinical Medicine, Trinity College Dublin, Dublin, Ireland; 9 Leeds Institute of Molecular Medicine, St. James’s University Hospital, University of Leeds, Leeds, United Kingdom; Queen Mary University of London, United Kingdom

## Abstract

**Objective:**

Iron deficiency (ID) and iron deficiency anaemia (IDA) are global major public health problems, particularly in developing countries. Whilst an association between *H. pylori* infection and ID/IDA has been proposed in the literature, currently there is no consensus. We studied the effects of *H. pylori* infection on ID/IDA in a cohort of children undergoing upper gastrointestinal endoscopy for upper abdominal pain in two developing and one developed country.

**Methods:**

In total 311 children (mean age 10.7±3.2 years) from Latin America - Belo Horizonte/Brazil (n = 125), Santiago/Chile (n = 105) - and London/UK (n = 81), were studied. Gastric and duodenal biopsies were obtained for evaluation of histology and *H. pylori* status and blood samples for parameters of ID/IDA.

**Results:**

The prevalence of *H. pylori* infection was 27.7% being significantly higher (p<0.001) in Latin America (35%) than in UK (7%). Multiple linear regression models revealed *H. pylori* infection as a significant predictor of low ferritin and haemoglobin concentrations in children from Latin-America. A negative correlation was observed between MCV (r = −0.26; p = 0.01) and MCH (r = −0.27; p = 0.01) values and the degree of antral chronic inflammation, and between MCH and the degree of corpus chronic (r = −0.29, p = 0.008) and active (r = −0.27, p = 0.002) inflammation.

**Conclusions:**

This study demonstrates that *H. pylori* infection in children influences the serum ferritin and haemoglobin concentrations, markers of early depletion of iron stores and anaemia respectively.

## Introduction

Iron deficiency (ID), the most common nutritional disorder in the world, and iron deficiency anaemia (IDA) affect 500–600 million people globally and represent a major public health problem particularly in developing countries [1], [2]. Factors including poor iron intake, low dietary iron bioavailability and gastrointestinal parasite infections contribute to the high frequency of ID/IDA in developing countries. Children are at risk as this age group has high iron requirements for growth [2]. In childhood, iron deficiency has been associated with deficits of immune, cognitive and motor function [2]. Clinically advanced IDA is associated with reduced growth, increased susceptibility to infectious diseases and increased mortality [3].

The high prevalence of combined *H. pylori* infection and ID/IDA in developing countries suggests that infection with this bacterium may be a cause of ID/IDA. Possible mechanisms include increased iron uptake by the *H. pylori* bacterium [4] and blood loss due to gastric lesions as a consequence of *H. pylori* infection [5]. Reduced iron absorption due to an elevated pH of gastric juice has also been attributed to *H. pylori* [6], [7] as there is transient hypochlorydria of variable duration in the early phase of infection and gastric atrophic changes in the late stages of infection [6], [8], [9]. As *H. pylori* infection is primarily acquired in childhood, and iron stores are lower in children than in adults, children are thought to be at a particular increased risk for iron deficiency.

Whereas some epidemiological studies and interventional trials have demonstrated evidence of an association between *H. pylori* infection and ID/IDA in children [10]–[13], in other studies this link has not been established [14]–[16]. In addition, studies evaluating children undergoing upper gastrointestinal endoscopy, which allows an accurate diagnosis of *H. pylori* infection as well as the exclusion of other common causes of ID such as coeliac disease and gastrointestinal bleeding, are scarce with few patients having been evaluated [17]–[18].

Therefore, the aim of this study was to evaluate the effects of *H. pylori* on iron deficiency in a large cohort of children (less than 16 years of age) with symptoms of dyspepsia undergoing upper gastrointestinal endoscopy.

## Patients and Methods

This study was approved by the Ethics Committee of the Universidade Federal de Minas Gerais, Belo Horizonte, Brazil; Pontificia Universidad Catolica de Chile, Santiago, Chile and East London Research Ethics Committee, United Kingdom (UK). The study was also reviewed by the European Union Ethics Committee. The trial was registered with the UK Clinical Research Network (Study Id 5149). Signed informed consent to participate was obtained from the children (whenever possible) and adolescents and their parents.

### Patients

The study cohort comprised 311 children (mean age 10.7±3.2 years, range 3–16 years, 179 girls) who were studied prospectively from June 2007 to September 2011 from three separate cities, Belo Horizonte and Santiago, in Latin America and London, UK. Belo Horizonte is located in Southeast Brazil, where the population shows a mixed ancestry, with well distributed contributions from Caucasian, African and Ameridians descent [19]. In Santiago, Chile, the population is composed by approximately two-thirds with European ancestry. Amerindians or persons of Amerindian admixture constitute most of the remaining third [20]. The children from London were born in England: 48% white; 32% south Asian; 10% black; and 10% mixed ethnic background. All children were symptomatic and underwent upper gastrointestinal endoscopy for investigation of symptoms referable to the upper gastrointestinal tract. The following patients were excluded: those who received antimicrobial drugs, anti-cholinergic and steroidal, non-steroidal anti-inflammatory agents and iron supplement for at least 30 days, proton pump inhibitors and H_2_-receptor antagonist for at least 15 days or antacid for 24 hours before endoscopy; those with peptic ulcer, coeliac disease and intestinal parasites; children with present or past history of gastrointestinal bleeding, oesophageal varices, coagulation disorders, inflammatory diseases, acquired or congenital immunosuppression, renal failure, hematologic disorders, neoplasia or an anatomical obstacle preventing endoscopy. From each female adolescent data was obtained on age of menarche, interval between the menses, duration and amount of monthly flow. Those with heavy menstrual blood loss were not included in the study. The menstrual cycle was considered normal when the interval between flows was 25–31 days and duration between 3 and 5 days [21]. In addition, after endoscopy, additional exclusion criteria were previously undiagnosed coeliac disease or any non-specific inflammation in the absence of duodenal gastric metaplasia. These criteria rigourously excluded possible confounding factors.

A detailed clinical history was obtained from each patient or their parents including presence (none, mild, moderate, severe) and duration (last week, last month, last year) of abdominal pain, acute or chronic diarrhoea, vomiting, heartburn as well as weight loss and fever and use of medications.

Clinical indication of endoscopy was classified according to the referral physician’s indication, as recurrent abdominal pain, gastroesophageal reflux disease, evaluation of vomiting, diarrhea or weight loss.

Biopsy specimens were obtained from antral, corpus and duodenal mucosa for histology and additional antral mucosal biopsies for evaluation of *H. pylori* status by culture and biopsy urease test.

Blood samples were collected from each patient and placed in sterile tubes with ethylenediamine tetraacetic acid to determine the full blood count and in iron-free tubes to determine the levels of serum ferritin, serum iron and serum total iron-binding capacity. IDA was defined as haemoglobin values lower than 110.0 g/L (children from 3 to 5 years of age), lower than 115.0 g/L (6 to 11 years of age) and lower than 120.0 g/L (12 to 16 years of age) and by serum ferritin concentration lower than 12 µg/L for children of 3 to 5 years of age and lower than 15 µg/L for those of 6 to 16 years of age.

Stool samples were obtained for parasitological assessment. Children with parasitic infection were excluded from analysis.

### *H. pylori* Status

*H. pylori* status was evaluated by culture, urease biopsy test and carbolfuchsin [22], Gimenez or Giemsa staining of histology sections. Patients were considered *H. pylori*-positive if the culture was positive or two other tests were positive and *H. pylori*-negative when the three tests were negative.

### Histology

Biopsies from the antral and corpus mucosa were fixed in 10% formalin and embedded in paraffin wax, and 4-µm-thick histological sections were stained with hematoxylin and eosin. The sections were analysed according to the revised Sydney System [23]. Active and chronic inflammation, intestinal metaplasia, atrophy and *H. pylori* density were graded as absent (0), mild (1), moderate (2), or marked (3). Sections of the duodenal mucosa were also assessed.

### Complete Blood Count

Blood counts were determined using automated electronic counters: Sysmex XT 1800i (Sysmex Corporation, Kobe, Japan) in Brazil, Beckman Coulter, model GeńS (Beckman Coulter Inc, CA, USA) in Chile and Sysmex XT 2100 (Sysmex Corporation, Kobe, Japan) in UK.

### Determination of Serum Ferritin, Serum Iron, Serum Total Iron-binding Capacity and Serum Transferrin Saturation

The serum concentration of ferritin was determined by a chemiluminescence method employing the ADVIA Centaur® Immunoassay CP System (Siemens Healthcare, Erlangen, Germany) in the samples from Brazil and Chile and an electrochemiluminescence immunoassay (Cobas Analyzer, Roche Diagnostics Ltd., Switzerland) in the samples from UK. The serum iron and the serum total iron binding capacity were determined by colorimetric methods (Brazil, Chile and UK). Serum transferrin saturation was obtained by dividing the serum iron concentration by the total iron binding capacity.

### Statistical Analysis

Data were analysed with SPSS statistical software package version 17.0 (SPSS Inc., Chicago, IL). In addition to the visual examination of the histograms and box plots, the Kolmogorov-Smirnov goodness-of-fit was used to assess the normality of the data. When significant departures from normality were detected, the data were log transformed. The degree of gastric active and chronic inflammation was compared among the groups by the two-tailed Mann Whitney U test. For other comparisons, the two-tailed Chi-square test, Fisher’s test or Student´s t test, Pearsońs or Spearman’s correlations were employed as indicated. The level of significance was set at p≤0.05. Multiple linear regression analyses (“enter option”) were used in order to quantify the simultaneous and mutually independent contribution of selected relevant predictor candidate, e.g. *H. pylori* infection, for low ferritin and haemoglobin blood concentration (dependent variables) while controlling for confounders such as gender and age. Variables with p values ≤0.20 in the univariate analyses were selected for the multivariate analyses. The optimum sample size, based on a significant level of 0.05 and a statistical power of 0.80 (type II error 0.02) for a multiple regression analyses with 4 predictor variables is at least 200 cases.

## Results

### Demographic and Clinical Characteristics of the Included Children

The demographic and clinical characteristics of the included children are described in Table 1. There was no statistically difference in regard to the age, gender and clinical indications of endoscopy among children from Belo Horizonte, Santiago and London (Table 1).

**Table 1:** Demographic and clinical characteristics of the included children according to the country of birth.

Variables	UK	Chile	Brazil	P value
	(n = 81)	(n = 105)	(n = 125)	
Mean age ± (SD)	10.1 (3.6)	10.6 (3.1)	11.1 (2.9)	0.58
Female/Male	46/35	59/46	74/51	0.88
Indications for endoscopy				
RAP (%)	65 (80.2)	84 (80.0)	102 (81.6)	0.95
GERD (%)	8 (9.9)	11 (10.5)	13 (10.4)	0.99
Vomiting (%)	3 (3.7)	9 (8.6)	4 (3.2)	0.14
Others* (%)	5 (6.2)	1 (0.9)	6 (4.8)	0.14
*H. pylori*-positivity (%)	6 (7.4)	33 (31.4)	47 (37.6)	<0.001

RAP, recurrent abdominal pain; GERD, gastroesophageal reflux disease; *diarrhea, weight loss.

### *H. pylori* Infection

The overall prevalence of *H. pylori* infection was 27·7% (86 children were *H. pylori*-positive and 225 *H. pylori*-negative). *H. pylori*-positive status was defined by positive culture in 71 (82.5%) children and by positive urease test and histology in 15 (17.5%) children.

The prevalence of infection was significantly higher in Santiago and Belo Horizonte than in London (p<0.001 for both) (Table 1), but no difference (p = 0.40) was observed when comparing Belo Horizonte and Santiago.

*H. pylori-*infected children (11.5±3.0 years) were older (p = 0.003) than those not infected (10.4±3.2 years), but no difference (p = 1.0) was observed between girls (27.4%, 49/179), and boys (28.0%, 37/132).

*H. pylori* infection was associated with vomiting (46.5% vs. 34.6% for infected and non-infected children, respectively; p = 0.05), but not with abdominal pain (89.5% vs. 85.3%; p = 0.29), chronic (5.8% vs. 10.2%; p = 0.32) or acute (9.3% vs. 15.1%, p = 0.25) diarrhoea, weight loss (18.6% vs. 22.2%; p = 0.59) and fever (9.3% vs. 8.0%; p = 0.89). When the population of each country was individually analysed, no association could be detected among the clinical symptoms and *H. pylori* infection in Belo Horizonte (p≥0.18) and London (p ≥ 0.58). However, in Santiago, *H. pylori* infection was associated with vomiting (41.9% *H. pylori*-positive vs. 24.2% *H. pylori*-negative; p = 0.05).

### Endoscopy and Histology

With respect to the gastric endoscopy findings, *H. pylori* infection was associated with the presence of antral (26.7% *H. pylori*-positive vs. 11.5% *H. pylori*-negative; p = 0.002) and corpus (8.1% *H. pylori*-positive vs. 0.9% *H. pylori*-negative; p = 0.002) erythema and antral nodularity (41.8% *H. pylori*-positive vs. 2.6% *H. pylori*-negative; p<0.001).

The degree of antral and corpus active and chronic inflammation was significantly higher (p<0.001 for all) in the *H. pylori*-positive than in *H. pylori*-negative children (Table 2). Antral lymphoid follicles were also more frequently observed (p<0.001) in infected (68.2%) than in the non-infected children (10.4%). Only two children presented with a degree of corpus atrophy. Neither antral atrophy nor antral and corpus intestinal metaplasia was observed.

**Table 2:** Histological comparison of antral (n = 85) and corpus (n = 83) gastric mucosa of *H. pylori (HP)*-positive children and antral (n = 202) and corpus (n = 214) gastric mucosa of *H. pylori*-negative children.*.

Inflammation	Absent	Mild	Moderate	Marked	P value
	n (%)	n (%)	n (%)	n (%)	
Antrum chronicinflammation					
HP-positive	2 (2.4)	32 (37.6)	50 (58.8)	1 (1.2)	
HP-negative	154 (76.2)	47 (23.4)	1 (0.4)	0	<0.001
Antrum activeinflammation					
HP-positive	13 (15.3)	44 (51.8)	27 (31.8)	1 (1.1)	
HP-negative	187 (92.6)	15 (7.4)	0	0	<0.001
Corpus chronicinflammation					
HP-positive	22 (26.5)	53 (63.9)	8 (9.6)	0	
HP-negative	156 (72.9)	57 (26.6)	1 (0.5)	0	<0.001
Corpus activeinflammation					
HP-positive	40 (48.2)	39 (46.9)	4 (4.9)	0	
HP-negative	191 (89.3)	22 (10.3)	1 (0.4)	0	<0.001

* Twenty-four antral and 13 corpus gastric biopsy specimens were deemed to be inadequate for histology assessment. n, number.

### IDA

IDA was detected in three *H. pylori*-positive children: a 13 year old girl from Belo Horizonte, with values of haemoglobin and serum ferritin of 111.0 g/L and 13.7 µg/L, respectively, and two girls from Santiago 14 and 12 years of age with haemoglobin and ferritin values of 119.0 g/L and 5.4 µg/L and 117.0 g/L and 10.0 µg/L, respectively, but no child uninfected with *H. pylori* had IDA (p = 0.02).

### Blood Iron Parameters

As significant departures from normality were detected for ferritin, concentrations were log-transformed and thus became normally distributed. The data obtained in the anaemia and iron parameters of the children from all countries according to *H. pylori* infection are presented in Table 3.

**Table 3:** Comparison of the iron deficiency/iron deficiency anaemia parameters between *H. pylori*-positive (n = 86) and negative (n = 225) children from Brazil, Chile and United Kingdom.

Variables	*H. pylori* status		P value
	Negative(mean±SD)	Positive (mean±SD)	
Serum ferritin (µg/L)	41.6 (29.9)	36.3 (23.5)	0.06
Transferrin saturation (%)*	29.2 (11.0)	28.3 (11.1)	0.52
Haemoglobin (g/L)	130.6 (9.6)	129.9 (10.9)	0.57
Haematocrit (L/L)	0.39 (0.03)	0.39 (0.03)	0.57
MCV (fL)	83.8 (4.7)	83.3 (5.0)	0.48
MCH (pg)	28.2 (1.8)	27.8 (1.9)	0.09

* obtained by dividing the serum iron concentration by the total iron binding capacity;

SD, standard deviation; MCV, mean corpuscular volume; MCH, mean corpuscular haemoglobin.

By analyzing all countries together by linear regression, *H. pylori* infection was not a predictor of low haemoglobin and ferritin, the best markers of iron deficiency and anemia, respectively (Table 4).

**Table 4:** Multiple linear regression models including ferritin or haemoglobin as dependent variables and age, gender, country of birth and *H. pylori* infection as independent variables in children from United Kingdom, Chile and Brazil (n = 311).

	Univariate analysis		Multivariate analysis	
	Beta	P value	Beta	P value
	coefficient		coefficient	
FERRITIN				
age	−0.12	0.84	–	–
gender	−0.203	<0.001	−0.204	<0.001
country of birth	−0.020	0.73	–	–
* H. pylori* infection	−0.108	0.06	−0.110	0.05
HAEMOGLOBIN				
age	0.303	0.000	0.327	<0.001
gender	−0.141	0.01	−0.194	<0.001
country of birth	−0.163	0.004	−0.141	0.009
* H. pylori* infection	−0.033	0.57	–	–

We then evaluated by linear regression each country separately (Table 5). *H. pylori* infection was not associated with low haemoglobin and low ferritin concentrations in London, but in Chile and in Brazil *H. pylori* infection was a predictor of low ferritin concentration. Furthermore, a tendency of association between the infection and low haemoglobin concentration was also observed in both countries.

**Table 5:** Multiple linear regression models including ferritin or haemoglobin as dependent variables and age, gender and *H. pylori* (HP) infection as independent variables in children from United Kingdom (n = 81), Chile (n = 105) and Brazil (n = 125).

	United Kingdom				Chile				Brazil			
	Univariateanalysis		Multivariateanalysis		Univariateanalysis		Multivariateanalysis		Univariateanalysis		Multivariateanalysis	
	Beta	P value	Beta	P value	Beta	P value	Beta	P value	Beta	P value	Beta	P value
	Coefficient		coefficient		coefficient		coefficient		coefficient		coefficient	
FERRITIN												
Age	−0.135	0.25	–	–	−0.046	0.64	–	–	−0.048	0.59	–	–
Gender	−0.157	0.02	–	–	−0.165	0.09	−0.169	0.08	−0.286	0.001	−0.276	0.02
HP infection	−0.043	0.71	–	–	−0.211	0.03	−0.214	0.03	−0.155	0.08	−0.197	0.05
HAEMOGLOBIN												
Age	0.451	<0.001	0.46	<0.001	0.187	0.06	0.294	0.005	0.280	0.002	0.304	0.001
Gender	−0.161	0.17	−0.199	0.07	−0.127	0.19	−0.248	0.01	−0.151	0.09	−0.140	0.10
HP infection	0.216	0.06	0.069	0.53	−0.182	0.06	−0.197	0.03	−0.164	0.09	−0.149	0.09

Taking into account the association observed between the iron parameters and *H. pylori* infection in the Latin-American countries in addition to the similar degree of socioeconomic development between the two populations, the results from Brazil and Chile were analysed together. In this combined population, *H. pylori* infection was an independent and significant predictor of low ferritin and haemoglobin concentrations. As expected, female gender was also a predictor of low ferritin and haemoglobin concentrations and increasing age was associated with increasing haemoglobin concentration (Table 6).

**Table 6:** Multiple linear regression models including ferritin or haemoglobin as dependent variables and age, gender, country of birth and *H. pylori* infection as independent variables in children from Chile (n = 105) and Brazil (n = 125).

	Univariate analysis		Multivariate analysis	
	Beta	P value	Beta	P value
	Coefficient		coefficient	
FERRITIN				
age	0.014	0.8	–	–
female	−0.222	0.001	−0.222	0.001
birth in Brazil	−0.155	0.02	−0.176	0.006
* H. pylori* infection	−0.164	0.01	−0.172	0.007
HAEMOGLOBIN				
age	0.236	<0.001	0.291	<0.001
female	−0.141	0.03	−0.191	0.003
birth in Brazil	−0.19	0.76	–	–
* H. pylori* infection	−0.130	0.04	−0.159	0.01

In these populations, but not in London children, *H. pylori* infection was also independently associated in the multivariate analysis with low values of haematocrit, MCV and MCH (Table S1 and Table S2).

### Gastric Pathology and Blood Iron Parameters

Inflammatory changes in the gastric mucosa induced by *H. pylori* are thought to contribute to changes in the gastric physiology which in turn influence iron absorption. We therefore evaluated the association between factors associated with reduced blood iron and gastric inflammatory parameters in the *H. pylori* infected and non-infected children.

In the *H. pylori*-positive children, a negative correlation was observed between MCV (r = −0.26; p = 0.01) and MCH (r = −0.27; p = 0.01) values and the degree of antral chronic inflammation; as well as between MCH and the degree of corpus chronic inflammation (r = −0.29, p = 0.008) and active inflammation (r = −0.27, p = 0.002). No other correlation was observed (p ≥ 0.19) in this group.

No correlations among the gastric inflammatory and the ID/IDA parameters were observed in the non-infected children (p>0.15).

## Discussion

Iron deficiency is the most common nutritional disorder in the world, particularly affecting children in developing countries. In addition to the high iron requirement in childhood, dietary iron bioavailability and gastrointestinal parasite infections are frequent in developing countries also contributing to iron deficiency in this age group.

This study, by analyzing children undergoing endoscopy for upper gastrointestinal symptoms that allowed better evaluation of *H. pylori* status and the exclusion of ID causes such as celiac disease, ulcer and erosions, demonstrates an inverse association between *H. pylori* infection and ferritin and haemoglobin concentrations, markers of iron deficiency and anaemia, respectively, in two Latin American countries. As ferritin is an acute phase protein that could, theoretically, be higher in the presence of *H. pylori* infection, the lower ferritin values we observed in the infected Latin American children points to the role of *H. pylori* in depletion of iron stores. Reinforcing experimentally these data, in an animal model, gastric *Helicobacter* infection leads to decrease in serum ferririn [25]. The mechanisms by which *H. pylori* might cause ID/IDA are still not determined; but, gastric acid is essential for iron absorption by reducing the ferric iron to a more soluble and absorbable ferrous iron form [2]. It has been previously demonstrated that *H. pylori* infected children have significantly lower basal and stimulated acid output and that eradication of *H. pylori* infection improved gastric acid output [26]. Furthermore, in the *H. pylori*-positive Chilean children, an association between low serum iron and transferrin and hypochlorhydria was observed [27]. Of note, in the Brazilian cohort, the concentration of gastric IL-1β, a potent inhibitor of gastric acid secretion, was inversely associated with blood ferritin and haemoglobin concentrations [28]. Consistent with these results, significant negative associations were also observed between the infection and other iron deficiency parameters: haematocrit, MCV and MCH. Furthermore, in this study we demonstrate for the first time negative associations between the severity of gastric inflammation and lower MCV and MCH values, which may represent decreased iron availability. This reinforces the hypothesis that the gastric physiological changes induced by *H. pylori* infection may be one mechanism that decreases iron absorption.

Conversely, in the United Kingdom cohort, the very low prevalence of *H. pylori* infection in an ethnically diverse group of children might explain the absence of association between the infection and iron deficiency/IDA parameters. A decreased prevalence of *H. pylori* infection associated with different generations of immigrants from developing to developed countries was similarly described by Tsai *et al*. [29] with Hispanics in the USA, likely reflecting a better standard of socioeconomic conditions. Thus, geographical variability among iron stores of the children may also explain the differences between the Latin American and UK cohorts we observed. Due to inadequate diet, children from developing countries could have a small iron reserve that contributes to the development of iron deficiency and IDA in the course of *H. pylori* infection.

Several studies describing associations between *H. pylori* infection and extra-gastric disease have been published. Among them, cross-sectional studies [13], [24] and clinical trials [12], [14] point to the role of *H. pylori* infection in the development of iron deficiency/IDA in children. However, there is considerable variation in the results of such studies largely due to methodological variation. Differences in the study design, inclusion criteria, number of included children, *H. pylori* diagnosis criterion and ethnicity could explain the discrepancies amongst the studies. It has to be emphasized that, in the present study, rigorous criteria in the selection of patients were adopted. Firstly, we studied only symptomatic children undergoing upper gastrointestinal endoscopy and biopsied gastric and duodenal mucosa. This allowed the exclusion of common causes of iron deficiency such as gastrointestinal bleeding, peptic ulcer disease, extensive erosions and celiac disease. Furthermore, in contrast to other studies [16], [30], female adolescents with heavy menstrual blood loss were not included because menstrual iron loss is an important determinant of iron status in young women. Intestinal parasitic infections that lead to blood loss were also an exclusion criterion. In addition, we included a large number of children and employed direct methods to diagnosis *H. pylori* infection. Most earlier studies used only one indirect test for diagnosis of *H. pylori* [14], [16], and these indirect tests, such as serology have a low accuracy rate for the diagnosis of *H. pylori* in children [31].

In conclusion, the results of this study demonstrate that *H. pylori* infection is a predictor of decreasing serum ferritin and haemoglobin concentrations. The gastric inflammation induced by the infection also negatively influences some haematological parameters. The last Maastricht Florence Consensus Report (Maastricht IV) recommends treating *H. pylori-*positive patients with IDA after the exclusion of the other common causes of the disease [32]. Based on the results of this study, children infected with *H. pylori* with decreased serum ferritin, even without anaemia, may well benefit from therapy for this microorganism.

## Supporting Information

Table S1 **Multiple linear regression models including haematocrit, mean corpuscular volume (MCV) and mean corpuscular haemoglobin (MCH) as dependent variables and age, gender, country of birth and** ***H. pylori*** **infection as independent variables in children from Chile (n = 105) and Brazil (n = 125).**
(DOC)

Table S2 **Multiple linear regression models including haematocrit, mean corpuscular volume (MCV) and mean corpuscular haemoglobin (MCH) as dependent variables and age, gender and** ***H. pylori*** **infection as independent variables in children from London (n = 81).**
(DOC)
