# Supplementary material for: Iron Status and Helicobacter pylori Infection in Symptomatic Children: An International Multi-Centered Study
Source: PLoS One. 2013 Jul 4;8(7):e68833. doi: 10.1371/journal.pone.0068833 (PMC3701645; doi:10.1371/journal.pone.0068833)
Supplement: Table S1 — Multiple linear regression models including haematocrit, mean corpuscular volume (MCV) and mean corpuscular haemoglobin (MCH) as dependent variables and age, gender, country of birth and H. pylori infection as independent variables in children from Chile (n = 105) and Brazil (n = 125). (DOC) [file pone.0068833.s001.doc]

**Table S1**- Multiple linear regression models including haematocrit, mean corpuscular volume (MCV) and mean corpuscular haemoglobin (MCH) as dependent variables and age, gender, country of birth and *H. pylori* infection as independent variables in children from Chile (n=105) and Brazil (n=125).

|  | Univariate analysis | |  |  | Multivariate analysis | |
| --- | --- | --- | --- | --- | --- | --- |
|  | Beta | P value |  | Beta | | P value |
|  | coefficient |  |  | coefficient | |  |
| HAEMATOCRIT |  |  |  |  | |  |
| age | 0.282 | <0.001 |  | 0.319 | | <0.001 |
| female | -0.096 | 0.015 |  | -0.155 | | 0.02 |
| birth in Brazil | 0.102 | 0.12 |  | 0.089 | | 0.16 |
| *H. pylori* infection | -0.088 | 0.19 |  | -0.128 | | 0.04 |
|  |  |  |  |  | |  |
| MCV |  |  |  |  | |  |
| age | 0.385 | <0.001 |  | 0.401 | | <0.001 |
| female | 0.086 | 0.19 |  | 0.027 | | 0.65 |
| birth in Brazil | -0.185 | 0.005 |  | -0.214 | | <0.001 |
| *H. pylori* infection | -0.119 | 0.07 |  | -0.153 | | 0.01 |
|  |  |  |  |  | |  |
| MHC |  |  |  |  | |  |
| age | 0.280 | <0.001 |  | 0.327 | | <0.001 |
| female | 0.013 | 0.84 |  | - | | - |
| birth in Brazil | -0.305 | <0.001 |  | -0.320 | | <0.001 |
| *H. pylori* infection | -0.151 | 0.02 |  | -0.165 | | 0.006 |
