# Supplementary material for: Iron Status and Helicobacter pylori Infection in Symptomatic Children: An International Multi-Centered Study
Source: PLoS One. 2013 Jul 4;8(7):e68833. doi: 10.1371/journal.pone.0068833 (PMC3701645; doi:10.1371/journal.pone.0068833)
Supplement: Table S2 — Multiple linear regression models including haematocrit, mean corpuscular volume (MCV) and mean corpuscular haemoglobin (MCH) as dependent variables and age, gender and H. pylori infection as independent variables in children from London (n = 81). (DOC) [file pone.0068833.s002.doc]

**Table S2**- Multiple linear regression models including haematocrit, mean corpuscular volume (MCV) and mean corpuscular haemoglobin (MCH) as dependent variables and age, gender and *H. pylori* infection as independent variables in children from London (n=81).

|  | Univariate analysis | |  |  | Multivariate analysis | |
| --- | --- | --- | --- | --- | --- | --- |
|  | Beta | P value |  | Beta | | P value |
|  | coefficient |  |  | coefficient | |  |
| HAEMATOCRIT |  |  |  |  | |  |
| age | 0.463 | <0.001 |  | 0.419 | | <0.001 |
| female | -0.194 | 0.10 |  | -0.213 | | 0.05 |
| *H. pylori* infection | 0.297 | 0.01 |  | 0.147 | | 0.20 |
|  |  |  |  |  | |  |
| MCV |  |  |  |  | |  |
| age | 0.235 | 0.04 |  | 0.201 | | 0.07 |
| female | 0.328 | 0.004 |  | 0.305 | | 0.007 |
| *H. pylori* infection | 0.019 | 0.87 |  | - | | - |
|  |  |  |  |  | |  |
| MHC |  |  |  |  | |  |
| age | 0.159 | 0.17 |  | 0.130 | | 0.25 |
| female | 0.265 | 0.02 |  | 0.250 | | 0.03 |
| *H. pylori* infection | -0.053 | 0.6 |  | - | | - |
